# Distribution of Primary Brain Tumor Subtypes in Lebanon: A Multicenter Eleven-Year Study of 695 Patients

**DOI:** 10.7759/cureus.17918

**Published:** 2021-09-13

**Authors:** Said El Hage, Mohamad Kawtharani, Sanaa Nabha, Jad El Masri, Mohamad Saad

**Affiliations:** 1 Faculty of Medical Sciences, Lebanese University, Hadath, LBN; 2 Neuroscience Research Center, Lebanese University, Beirut, LBN; 3 Neuroscience, Lebanese University, Beirut, LBN; 4 Qatar Computing Research Institute, Hamad Bin Khalifa University, Doha, QAT

**Keywords:** glioblastoma, meningioma, lebanon, epidemiology, brain neoplasms

## Abstract

Background

Brain tumors are associated with relatively high mortality and morbidity in comparison with their low incidence. Little is known about primary brain tumors in Lebanon, as well as in the Arab world. This study aims to analyze the epidemiology of brain tumors across the Lebanese population.

Methods

Data from pathology reports of patients diagnosed with malignant and non-malignant primary brain tumors were collected retrospectively in an eleven-year period (2007-2017) from four medical centers in Lebanon. A total of 695 primary brain tumor cases (61% malignant and 39% non-malignant) were retrieved from different regions across the country.

Results

Meningiomas were the most common histology in this sample (29.6%), followed by glioblastomas (25.5%) and oligodendrogliomas (5.9%). Pituitary tumors were only 3.5% of brain tumors. Besides, the most common anatomical locations in malignant and non-malignant tumors were cerebral meninges (29.6%), the "other brain" category (21.3%), and the frontal lobe (11.2%). In children and adolescents, embryonal tumors (21%) were the most common histologies, while glioblastomas and meningiomas accounted for 14.8% and 13.6%, respectively.

Conclusion

Lebanon presented a low rate of pituitary tumors and an unusually high percentage of malignant tumors, as well as pediatric glioblastomas and meningiomas. This should raise major concerns for policymakers to detect the possible underlying causes.

## Introduction

According to the World Cancer Research Fund, brain and other central nervous system (CNS) tumors are ranked the 17th most common cancer in the world and account for 1.7% of all cancer, excluding non-melanoma skin cancer [1]. Patel et al. reported a 17.3% increase in central nervous system cancer between 1990 and 2016. Along with this surge in incident cases and despite their rarity, CNS tumors represent a disproportionally high source of morbidity and mortality worldwide [2,3]. These tumors cause a high burden on societies and health care systems because of their substantial malignant potential and the cost of the complex treatment required, ranging from chemotherapy, radiation therapy, and neurosurgery. However, the incidence varies significantly between studies, probably due to the lack of a standardized approach to quantify the results [4]. Brain tumors are the most common solid tumors in the pediatric population. Compared to adults, pediatric brain tumors have different histological distribution and are most commonly located in the infratentorial location [5,6].

Brain tumors encompass more than 100 different histologies, classified into more than 20 major histological groups [7,8]. To date, the causes of brain tumors remain unclear, with only a small proportion caused by radiation, immunosuppression leading to brain lymphomas, and hereditary genetic syndromes as in neurofibromatosis, Li-Fraumeni syndrome, and Turcot syndrome [9]. Also, there is a predilection for some brain tumor subtypes with certain risk factors. For instance, meningioma is tightly associated with previous radiation exposure and is more common among females [9].

In the Middle East region, few types of research have addressed the issue of brain tumors. Most papers describing the epidemiology of these tumors were from Saudi Arabia, Egypt, and Kuwait [10-13]. For instance, a study in Western Saudi Arabia found that the most common brain tumor was astrocytoma and that their results were similar to international ranges [11]. Another study conducted in Egypt showed that gliomas followed by meningiomas were the most common histologies [13]. Moreover, in Lebanon, a small country in the Middle Eastern region with a population of nearly 6.8 million people, 238 cases of brain cancers were reported in 2016 by the Ministry of Public Health (MOPH) [14]. To date, no study has been conducted to describe the epidemiology of the different types of brain tumors among its population. This paper outlines the distribution of these tumors, in four Lebanese medical centers, concerning age, sex, behavior, histology, and anatomical location. Our objective is to show the most common histologies in our sample and provide a detailed report of the different malignant and non-malignant tumors. We also compare our results with available data from other countries in the region and worldwide. 

## Materials and methods

Study design

This is an epidemiological descriptive retrospective study in which data were collected from pathology reports of 695 patients diagnosed with primary brain tumors in the period between January 2007 and July 2017. Secondary (metastatic) brain tumors that rose primarily in a site outside of the CNS and tumors arising from the spinal cord were excluded from this study. Only primary brain tumors from any age were included. Data were retrieved from four medical centers across Lebanon: Institut National de Pathologie (INP) in Hadath; Hammoud Hospital University Medical Center (HHUMC) in South Lebanon; Al Zahraa Hospital University Medical Center (ZHUMC) in Beirut; and Sahel General Hospital (SGH) in Beirut. INP is a referral center of pathology for more than 30 centers and hospitals across Lebanon, including Abou Jaoude Hospital, Bekaa Hospital, Ain Wazein hospital, and others (Saad et al.) [15]. This study was approved by the Institutional Review Boards (IRB) of all four previously mentioned hospitals, as well as the Lebanese University’s IRB.

Variable coding

The collected variables were sex, age at diagnosis, histopathological type of the tumor, anatomical location (frontal lobe, occipital lobe, temporal, meninges, etc.), and behavior (malignant vs. non-malignant). Histology, behavior, and anatomical location were coded according to the International Classification of Diseases of Oncology, third edition (ICD-O-3) manual, and in line with the manual’s coding guidelines for both topography and morphology, as well as 2007 WHO classification for the CNS, which was used especially for behavior coding [7,8].

Moreover, Ostrom et al. used a grouping of sites that is based on the WHO ICD-O-3 classification of oncology [7,8]. This grouping has been used in this report to classify anatomical sites and histologies of these tumors. The topographical areas and assigned codes that were used in this report can be found in the appendix (Table 1). All histologies and assigned ICD-O-3 histology codes and behaviors reported in this study are also shown in the appendix (Table 2).

Although pilocytic astrocytoma is coded as non-malignant by the WHO code, it is considered malignant in population-based cancer registries in the United States [8]. Therefore, to have comparable data, we coded pilocytic astrocytoma as malignant (appendix, Table 2). Besides, some tumors in our report were labeled “low-grade gliomas” and were coded as 9380/1, translated as gliomas with uncertain malignant or benign behavior. The use of this code is also supported by Percy et al. [16].

Statistical analysis

Descriptive statistics were conducted using SPSS (Statistical Package for Social Sciences) version 23.0 statistical analysis software (IBM Inc., Armonk, New York). Duplicates between centers were checked and subsequently removed. Basic descriptive analysis is conducted for categorical and continuous variables (bar graphs, pie charts and scatter plots). Chi-square tests were used for categorical variables, and a chi-square for homogeneity test was used for testing whether the proportions of variable categories are equal.

## Results

Overview

Table 3 in the appendix depicts in detail the different histologies found in our dataset, as well as the different sex, behavior, and age groups. Over 40 different histologies were found in our sample. Most of the patients were from INP 70.36% (489 patients), 13.38% (93 patients) from HHUMC, 11.08% (77 patients) from ZHUMC, and 5.18% (36 patients) from SGH. Interestingly, 61% of the tumors were of malignant behavior, while only 39% were non-malignant. Tumors ranged from common tumors such as meningiomas (29.6%) to rare histologies such as dysembryoplastic neuroepithelial tumor (0.1%), atypical teratoid rhabdoid tumor (0.1%), and solitary fibrous tumor (0.1%).

Anatomical location

**Figure 1:**
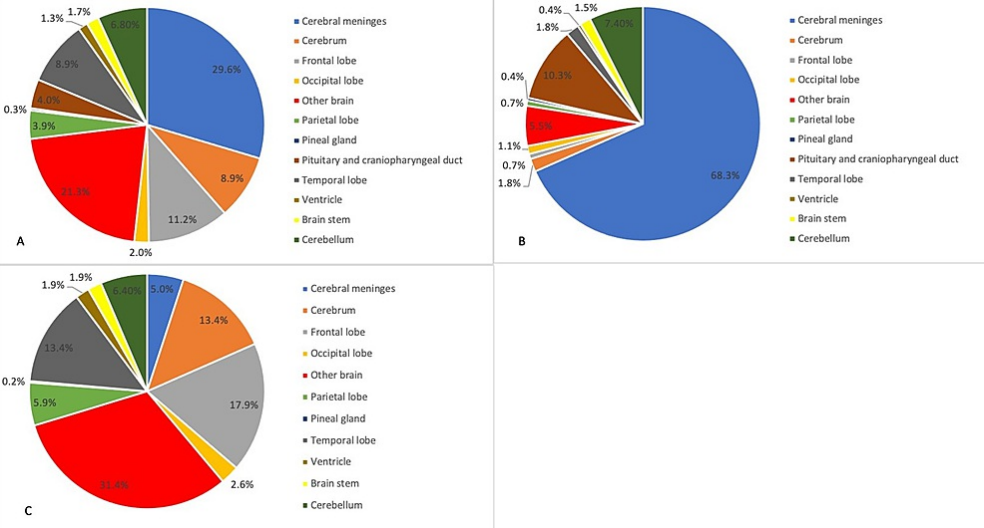
Distribution of primary brain tumors by anatomical location and behavior A) overall (N=695), B) non-malignant (N=271), C) malignant (N=424)

Figure 1 shows the distribution of brain tumors by anatomical location. Overall, the most common locations were the cerebral meninges (29.6%); "other brain" category, which encompasses overlapping regions in the brain, and unspecified brain locations (21.3%); and the frontal lobe (11.2%). Only 1.3% of brain tumors were found in the ventricles and 1.7% in the brainstem (Figure 1A).

Among non-malignant brain tumors, the most common location was cerebral meninges with a frequency of 68.3%, followed by the pituitary and craniopharyngeal duct (10.3%) and cerebellum (7.4%) (Figure 1B). Among malignant cases, our results showed that "other brain" category was the most common location (31.4%), followed by the frontal lobe (17.9%) and temporal lobe (13.4%) (Figure 1C).

Histology Distribution

**Figure 2:**
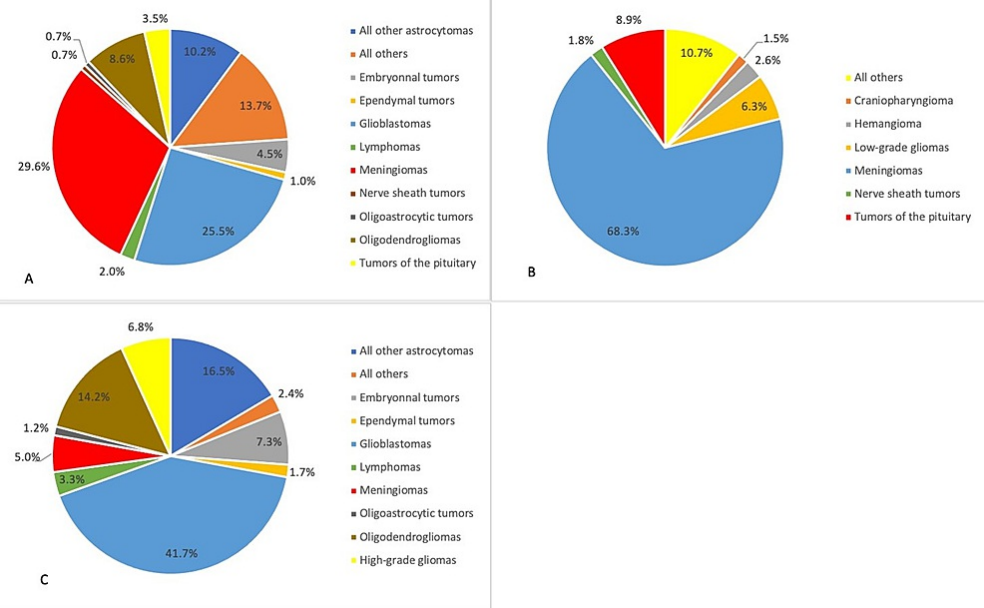
Distribution of primary brain tumors by histology and behavior A) overall (N=695), B) non-malignant (N=271), C) malignant (N=424) All other astrocytomas include pilocytic astrocytoma, diffuse astrocytoma, anaplastic astrocytoma, and unique astrocytoma variants. Oligodendrogliomas includes oligodendroglioma and anaplastic oligodendroglioma.

Histologies in our sample were organized using the Central Brain Tumor Registry of the United States (CBTRUS) groups, as mentioned earlier. Figure 2 represents the distribution of brain tumor histologies. Overall, meningiomas were the most common histology in our sample with 206 cases (29.6%). Glioblastomas were the second most common histology with 177 cases (25.5%). Nerve sheath tumors and ependymal tumors were found to be rare, accounting for only 0.7% and 1% of the total number of cases, respectively (Figure 2A). Among malignant cases, glioblastomas were most commonly found (41.7%), followed by all other astrocytomas (16.5%), oligodendrogliomas (14.2%), and embryonal tumors (7.3%) (Figure 2C). Among non-malignant cases, meningiomas were the most common histology with a frequency of 68.3%, while tumors of the pituitary, craniopharyngioma, and hemangioma accounted for 8.9%, 1.5%, and 2.6%, respectively (Figure 2B).

Sex distribution

To analyze the distribution of sex, the male to female ratio was calculated and the chi-square test for homogeneity was used for determining whether the proportions of males and females are equal. Except for non-malignant meningiomas, chi-square tests revealed that males and females had an equal distribution of brain tumors (p >0.05). Non-malignant meningiomas were 2.6 times more common among women than men (male-to-female ratio = 0.38, p <0.001).

Distribution by age groups

**Figure 3:**
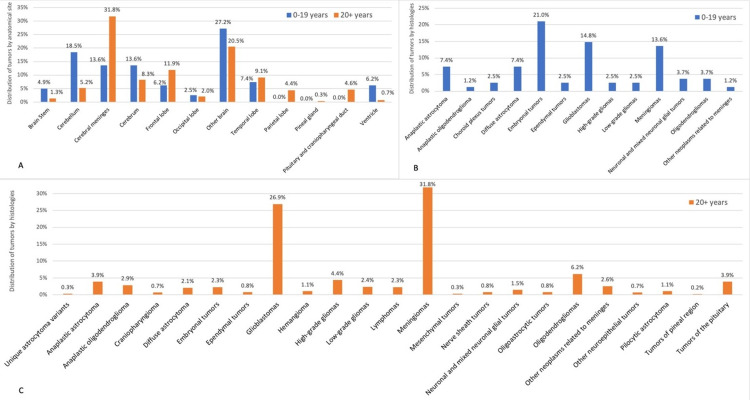
Distribution of primary brain tumors A) children and adolescents (0-19 years) (N=81) and (20+ years) (N=614)  by anatomical location, B) children and adolescents (0-19 years) by histologies (N=81), and C) adults (20+ years) by histologies (N=614)

**Figure 4:**
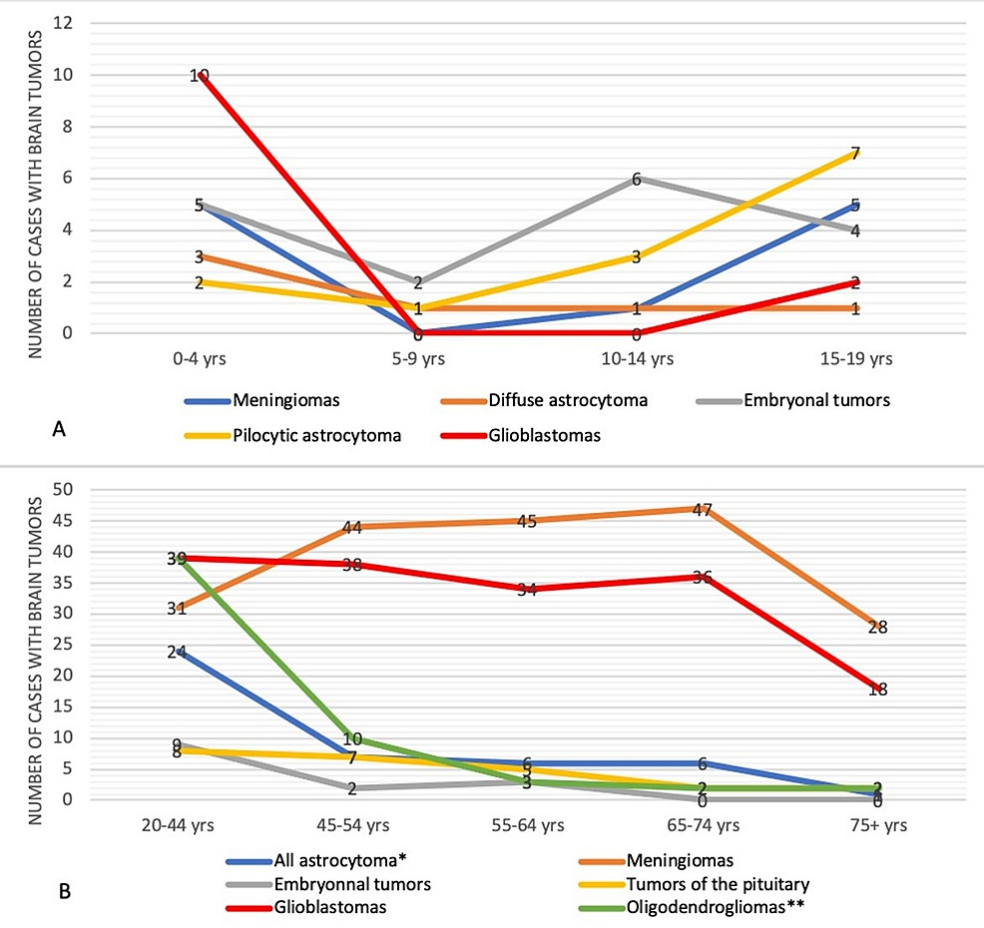
Distribution of brain tumor cases by age A) age 0-19 years B) age 20+ years * All other astrocytoma includes pilocytic astrocytoma, diffuse astrocytoma, and anaplastic astrocytoma ** Oligodendrogliomas include oligodendrogliomas (ICD-O-3 code 9450) and anaplastic oligodendrogliomas (ICD-O-3 code 9451)

The mean age of diagnosis of brain tumors was 47.43 years (SD= 21.20). Table 3 in the appendix details the distribution of tumors according to three age groups (0-14 years, 15-39 years, and 40+ years). However, to simplify the analysis, we used two main age groups (0-19 years for children and adolescents and 20+ years for adults) in subsequent pie charts and plots (e.g., Figures 3-4).

Our results showed that brain tumors were significantly higher in adults than in children and adolescents (88.34%, 614 cases, vs. 11.65%, 81 cases; p <0.001). Also, the ratio of malignant-to-non-malignant cases in children and adolescents was significantly higher compared to adults (4.06 vs. 1.41, p <0.001). As a result, children and adolescents had a higher risk of being diagnosed with malignant brain tumors compared to adults.

Figure 3 shows the distribution of brain tumors by site and histology in each age group. The most common location of brain tumors in children and adolescents was "other brain" (27.2%), while cerebral meninges was the most common location in adults(31.8%). Tumors in the cerebellum and brain stem were more prevalent in children and adolescents (18.5% and 4.9%, respectively), in comparison to adults (5.2% and 1.3%, respectively) (Figure 3A). Embryonal tumors such as medulloblastoma were the most common reported histologies (21.0%) in children and adolescents, followed by pilocytic astrocytoma (16.0%) and interestingly by glioblastomas (14.8%) and meningiomas (13.6%) (Figure 3B). In contrast, the most common tumors in adults were meningiomas (31.8%), glioblastomas (26.9%), and oligodendrogliomas (6.2%). On the other hand, in adults, miscellaneous tumors such as pilocytic astrocytoma, ependymal tumors, nerve sheath tumors, and craniopharyngioma were found at rates close to 1% (Figure 3C).

A more robust analysis of selected tumors, as shown in Figure 4, shows their distribution across multiple fine-grained age groups. Surprisingly, 10 cases of glioblastomas and 5 cases of meningiomas were diagnosed between 0-4 years (specifically at age 0), then abruptly decreasing till age 10-14 years. These tumors tend to resurge and stabilize between 45 to 74 years, with a peak of 47 cases in the 65-74 age group. Furthermore, pilocytic astrocytoma reached a peak of seven cases at 15-19 years in children and adolescents. The most conspicuous observation in children and adolescents is that most tumors diagnosed in ages 5-9 years had the lowest number of cases, compared to other age groups. However, in adults, a high number of cases of oligodendrogliomas (39 cases) and all other astrocytomas (24 cases) were found in younger adults (20-44 years). Tumors of the pituitary were found in stable numbers, with approximately seven cases per class of age, between ages 20 till 64 years of age (Figure 4B). Besides, the highest number of cases of embryonal tumors was reported in the 20-44 age group (nine cases), followed by seven cases in the 15-19 age group (Figure 4).

## Discussion

Strikingly, in the CBTRUS database, only 30.9% of cases were malignant, and 69.1% were non-malignant [8]. However, in our Lebanese cohort, malignant tumors were approximately twice more common than nonmalignant tumors (61% malignant and 39% non-malignant). This should raise major concerns and in-depth studies to reveal the cause of this high malignancy in the Lebanese population. Further research should be conducted, especially to assess risk factors such as radiation exposure and possible diagnosis of hereditary cancer syndromes. Clinicians in Lebanon should be highly suspicious in case of brain tumors due to the high malignancy rates.

In comparison with the CBTRUS database between 2011 and 2015, anatomical locations were relatively similar [8]. Cerebral meninges, as well as meningioma, were the most common anatomical location and histological subtype in non-malignant tumors in both databases (approximately 53.0% in CBTRUS vs. 68.3% in Lebanon). This indicates that meningiomas, which have a favorable prognosis, are the leading non-malignant tumors in Lebanon. Among malignant tumors, the frontal lobe and other brain categories were the most common locations (23.9% and 22.5% in CBTRUS vs. 17.9% and 31.4% in Lebanon, respectively). Interestingly, the pituitary and craniopharyngeal duct accounted for 17.5% of the overall anatomical location in the CBTRUS database while accounting for only 4.0% of tumors in our study. This could indicate a low incidence of pituitary tumors and craniopharyngioma (3.5% and 0.4%, respectively) in Lebanon, a lack of equipment in diagnosing these tumors, a lack of awareness by physicians of pituitary pathologies, and the referral of patients with these types of tumors to other medical centers not included in our study. Therefore, further evaluation is needed to strengthen these findings [17].

When comparing percentages of brain tumors between randomly selected countries in the Middle East and the West, such as Iran and Jordan [12,17], as well as Austria (the Austrian Brain Tumor Registry) and the United States [8,18], results were quite similar for astrocytomas, meningiomas, medulloblastoma, and ependymoma. However, in comparison with the previously mentioned countries, Lebanon presented the highest rates of glioblastomas, grade IV tumors that are highly malignant (25.47% in Lebanon vs. 14.7% in the United States, 20.1% in Austria, and 18.9% in Jordan), and the lowest rates of tumors of the pituitary and nerve sheath tumors (mostly schwannomas), which are predominantly non-malignant (3.5% and 0.7%, respectively, vs. 16.3% and 8.6% in the United States).

Sex distribution showed significant results in non-malignant meningiomas, which was more prevalent in females than males. It is well established that females are at a higher risk of developing meningiomas [8]. The non-significant results seen in other tumors could be due to the small number of cases or nonspecific sex distribution.

Glioblastoma and meningioma are rare entities in the pediatric population. In general, these tumors occur mostly in adults. In the United States, for example, glioblastomas accounted for 3.1% and meningiomas for 2.7% of tumors in children and adolescents (0-19 years) [8]. In Syria, meningiomas accounted for 1.1% of childhood brain tumors between the years 2002-2008 [19]. Strikingly, our results showed that these tumors had higher rates than usual. In Lebanon, meningiomas accounted for 13.6% (11 cases) and glioblastomas for 14.8% (12 cases) of tumors in children and adolescents (0-19 years). Also, 2.5% of tumors in this sample were reported as high-grade gliomas not otherwise specified (NOS), referring to grade III and IV tumors, including glioblastomas.

Further analysis, as depicted in Figure 4A, shows that 10 out of 12 cases of pediatric glioblastomas occurred between 0-4 years (more specifically at age 0), and 5 out of 11 cases of pediatric meningiomas occurred between 0-4 years. Among these five cases of meningiomas, four cases occurred during the first 12 months of age, and one case occurred at three years of age; all were diagnosed between 2010 and 2015. They were located in the cerebral meninges; three cases were females, and two were males. A literature review on meningiomas in neonates reveals that these tumors are usually cystic and are not significantly higher in females, in opposition to adults [20]. Contrary to our results, large series show that intracranial meningiomas account only for 0.4-4.6% of all brain tumors in children (Mehta et al.) [21]. Therefore, a case report of these five cases of meningiomas, found in our sample, should be conducted to find the causes, clinical presentations as well as more detailed epidemiology to untie the knot of these rare entities.

As for congenital glioblastomas, less than 50 cases have been reported in the literature and were the rarest congenital brain tumors [22]. In our sample, 10 cases of glioblastoma multiforme (GBM) occurred in the first year of life between 2007 and 2015, with an equal sex distribution. Five cases were located in the cerebrum, two cases in the temporal lobe, one case in the frontal lobe, and two cases were in the brain, NOS. We speculate that the causes of these findings could be either genetic or hereditary causes, exposure to radiation, or possible misdiagnosis. Barbour et al. found that Lebanon has a high prevalence of consanguineous marriages (35.5%), which could lead to a high incidence of hereditary and genetic syndromes [23]. In support of this theory, a study in Saudi Arabia found that approximately 40% of 1742 children diagnosed with cancer had hereditary cancer susceptibility, and consanguinity was reported in 38% of these patients [24]. Nevertheless, Lebanon, a politically and economically unstable country, witnessed many wars throughout history, such as the 1975 Civil War, the Israeli occupation between 1982 and 2000, the 2006 Israeli Lebanese war, and many other conflicts, which could result in possible radiation exposure [25]. Also, a study in California on the ethnic Middle Eastern population found that this particular population had a higher incidence of benign meningiomas and speculated that one of the risk factors could be a previous childhood radiation exposure from Israel [26]. In fact, the incidence of meningioma was found to be high among Hiroshima atomic bomb survivors [27]. This could explain the high number of pediatric meningiomas and glioblastomas found in our sample. Furthermore, Merabi et al. assessed the importance of a second opinion in diagnosing pathology samples in children’s cancers [28]. Conducted in Lebanon and reviewing more than 171 pathology reports of different childhood cancers, the authors found that 71% of disagreements occurred in pediatric brain tumors. Therefore, in the topic of childhood brain tumors, we suggest using a second opinion, preferably a specialized neuropathologist’s opinion, as well as using novel molecular diagnostics to increase the accuracy of diagnosis and avoid misdiagnosis. Moreover, 16 cases (2.3%) of hemangioblastomas were reported in our sample. They were all present in the cerebellum. Interestingly, one-third of hemangioblastomas are associated with Von Hippel Lindau (VHL) syndrome [29]. Meningiomas, glioblastomas as well as other gliomas can be associated with genetic syndromes [9]. Genetic risk should be assessed in future research to establish the links with brain tumors in the Lebanese population. Environmental factors such as pesticide exposure in farmers, which is prevalent in Lebanon, can increase the risk of brain tumors by 1.3 to 3.6 fold [30].

This study could not gather all the cases of brain tumors in Lebanon due to the variety of hospitals treating these tumors. Therefore, incidence rates could not be calculated, and age standardization was not feasible. Data about the laterality of tumors (left/right), the geographical distribution of brain tumors, and ethnic groups could not be gathered. These variables could be useful for a more in-depth analysis.

## Conclusions

Little is known about the distribution of brain tumors in Lebanon. The aim of this study was to present comprehensive epidemiology of primary brain tumors in the Lebanese population. Our study revealed a very high percentage of malignant brain tumors; new policies should be implemented to improve research in this field and to ensure better screening and awareness. Further investigation is needed to evaluate the possible causes of the high incidence of pediatric glioblastomas and meningiomas in the Lebanese population.

## Appendices

**Table 1:** Brain tumor anatomical groups NOS - not otherwise specified

Site	ICD-O-3 Site Code
Cerebrum	C71.0
Frontal lobe	C71.1
Temporal lobe	C71.2
Parietal lobe	C71.3
Occipital lobe	C71.4
Ventricle	C71.5
Cerebellum	C71.6
Brain Stem	C71.7
Other brain	
Overlapping lesion of brain	C71.8
Brain, NOS	C71.9
Cerebral meninges	C70.0
Pituitary and craniopharyngeal duct	
Pituitary gland	C75.1
Craniopharyngeal duct	C75.2
Pineal gland	C75.3

**Table 2:** ICD-O-3 Histological coding of brain tumors found in our study

		ICD-O-3 Histology and behavior code		
Histology	ICD-O-3 Histology code	Malignant (ICD-O-3 behavior /3)	Non-malignant (ICD-O-3 behavior /0 and /1)	
Tumors of neuroepithelial tissue				
Pilocytic astrocytoma	9421	9421/1	-	
Diffuse astrocytoma	9400	9400/3	-	
Anaplastic astrocytoma	9401	9401/3	-	
Unique astrocytoma variants	9424	9424/3	-	
Glioblastoma	9440, 9442	9440/3, 9442/3	-	
Oligodendroglioma	9450	9450/3	-	
Anaplastic oligodendroglioma	9451	9451/3	-	
Oligoastrocytic tumors	9382	9382/3	-	
Ependymal tumors	9383, 9391, 9392	9391/3, 9392/3	9383/1	
High-grade glioma	9380	9380/3	-	
Low-grade glioma	9380	-	9380/1	
Choroid plexus tumors	9390	9390/3	-	
Other neuroepithelial tumors	9430	9430/3	-	
Neuronal and mixed neuronal-glial tumors	8680, 9413, 9505, 9506	9505/3	8680/0.1, 9413/0, 9505/1, 9506/1	
				Tumors of the pineal region	9360-9362	9362/3	9360/1, 9361/1	
Embryonal tumors	9470-9474, 9500-9502, 9508	9470/3, 9471/3, 9472/3,9473/3, 9474/3, 9501/3, 9502/3, 9503/3, 9508/3	-	
				Tumors of cranial and spinal nerves				
Nerve sheath tumors	9560	-	9560/0.1	
Tumors of Meninges				
Meningioma	9530, 9539	9530/3	9530/0.1, 9539/1	
Mesenchymal tumors	8815, 9260	9260/3	-	
Other neoplasms related to meninges	9161, 9370	9370/3	9161/1	
Lymphomas and hematopoietic neoplasms				
Lymphoma		9590/3	-	
Tumors of sellar region				
Tumors of the pituitary	8272	-	8272/0	
Craniopharyngioma	9350, 9352	-	9350/1, 9352/1	
Unclassified tumors		-		
Hemangioma	9120, 9121, 9133	-	9120/0, 9121/0, 9133/1	

**Table 3:** Distribution of all histologies by sex, age, and behavior * Percentages are out of the total sample (N=695) and may not add up to 100% because of rounding. cPNET - central primitive neuroectodermal tumor

	Male	Female	Malignant	Non-malignant	0 till 14 years	15 till 39 years	40+ years	Total *
Tumors of neuroepithelial tissue								
Pilocytic astrocytoma	11 (55%)	9 (45%)	20 (100%)	0 (0%)	6 (30%)	12 (60%)	2 (10%)	20 (2.9%)
Diffuse astrocytoma	11 (57.9%)	8 (42.1%)	19 (100%)	0 (0%)	5 (26.32%)	9 (47.36%)	5 (26.32%)	19 (2.7%)
Anaplastic astrocytoma	17 (56.7%)	13 (43.3%)	30 (100%)	0 (0%)	5 (16.67%)	7 (23.33%)	18 (60%)	30 (4.3%)
Unique astrocytoma variants:								
Xanthoastrocytoma	0 (0%)	1 (100%)	1 (100%)	0 (0%)	0 (0%)	0 (0%)	1 (100%)	1 (0.14%)
Glioblastoma								
Glioblastoma multiforme	82 (49.7%)	83 (50.3%)	165 (100%)	0 (0%)	10 (6.06%)	17 (10.30%)	138 (83.64%)	165 (23.7%)
Gliosarcoma	4 (33.3%)	8 (66.7%)	12 (100%)	0 (0%)	0 (0%)	3 (25%)	9 (75%)	12 (1.7%)
Oligodendrogliomas	26 (63.4%)	15 (36.6%)	41 (100%)	0 (0%)	1 (2.44%)	24 (58.54%)	16 (39.02%)	41 (5.9%)
Anaplastic oligodendroglioma	11 (57.9%)	8 (42.1%)	19 (100%)	0 (0%)	1 (5.26%)	7 (36.84%)	11 (57.90%)	19 (2.7%)
Oligoastrocytic tumors	3 (60%)	2 (40%)	5 (100%)	0 (0%)	0 (0%)	2 (40%)	3 (60%)	5 (0.7%)
Ependymal tumors								
Subependymoma	0 (0%)	1 (100%)	0 (0%)	1 (100%)	0 (0%)	0 (0%)	1 (100%)	1 (0.1%)
Ependymoma	2 (50%)	2 (50%)	4 (100%)	0 (0%)	1 (25%)	2 (50%)	1 (25%)	4 (0.6%)
Anaplastic ependymoma	0 (0%)	3 (100%)	3 (100%)	0 (0%)	1 (33.33%)	1 (33.33%)	1 (33.33%)	3 (0.4%)
High grade glioma	18 (62.1%)	11 (37.9%)	29 (100%)	0 (0%)	2 (6.90%)	5 (17.24%)	22 (75.86%)	29 (4.2%)
Low grade glioma	8 (47.1%)	9 (52.9%)	0 (0%)	17 (100%)	2 (11.76%)	5 (29.42%)	10 (58.82%)	17 (2.4%)
Choroid plexus tumors	2 (100%)	0 (0%)	2 (100%)	0 (0%)	2 (100%)	0 (0%)	0 (0%)	2 (0.3%)
Other neuroepithelial tumors								
Astroblastoma	3 (75%)	1 (25%)	4 (100%)	0 (0%)	0 (0%)	2 (50%)	2 (50%)	4 (0.6%)
Neuronal and mixed neuronal-glial tumors								
Paraganglioma	0 (0%)	1 (100%)	0 (0%)	1 (100%)	0 (0%)	0 (0%)	1 (100%)	1 (0.1%)
Dysembryoplastic neuroepithelial tumor	1 (100%)	0 (0%)	0 (0%)	1 (100%)	0 (0%)	1 (100%)	0 (0%)	1 (0.1%)
Ganglioglioma	1 (25%)	3 (75%)	1 (25%)	3 (75%)	0 (0%)	3 (75%)	1 (25%)	4 (0.6%)
Central neurocytoma	2 (33.30%)	4 (66.7%)	0 (0%)	6 (100%)	3 (50%)	1 (16.67%)	2 (33.33%)	6 (0.9%)
Tumors of the pineal region	1 (100%)	0 (0%)	0 (0%)	1 (100%)	0 (0%)	1 (100%)	0 (0%)	1 (0.1%)
Embryonal tumors								
Medulloblastoma	12 (48%)	13 (52%)	25 (100%)	0 (0%)	9 (36%)	12 (48%)	4 (16%)	25 (3.5%)
Desmoplastic medulloblastoma	0 (0%)	1 (100%)	1 (100%)	0 (0%)	1 (100%)	0 (0%)	0 (0%)	1 (0.1%)
cPNET	0 (0%)	1 (100%)	1 (100%)	0 (0%)	0 (0%)	0 (0%)	1 (100%)	1 (0.1%)
Neuroblastoma	1 (100%)	0 (0%)	1 (100%)	0 (0%)	0 (0%)	0 (0%)	1 (100%)	1 (0.1%)
Medulloepithelioma	0 (0%)	2 (100%)	2 (100%)	0 (0%)	2 (100%)	0 (0%)	0 (0%)	2 (0.3%)
Atypical teratoid rhabdoid tumor	0 (0%)	1 (25%)	1 (100%)	0 (0%)	1 (100%)	0 (0%)	0 (0%)	1 (0.1%)
Tumors of cranial and spinal nerves								
Schwannoma	3 (60%)	2 (40%)	0 (0%)	5 (100%)	0 (0%)	3 (60%)	2 (40%)	5 (0.7%)
Tumors of Meninges								
Meningioma	63 (30.58%)	143 (69.42%)	21 (10.19%)	185 (89.81%)	6 (2.92%)	19 (9.22%)	181 (87.86%)	206 (29.6%)
Mesenchymal tumors								
Solitary fibrous tumor	0 (0%)	1 (100%)	1 (100%)	0 (0%)	0 (0%)	1 (100%)	0 (0%)	1 (0.1%)
Ewing sarcoma	0 (0%)	1 (100%)	1 (100%)	0 (0%)	0 (0%)	1 (100%)	0 (0%)	1 (0.1%)
Other neoplasms related to meninges								
Hemangioblastoma	10 (62.5%)	6 (37.5%)	0 (0%)	16 (100%)	1 (6.25%)	6 (37.5%)	9 (56.25%)	16 (2.3%)
Chordoma	1 (100%)	0 (0%)	1 (100%)	0 (0%)	0 (0%)	1 (100%)	0 (0%)	1 (0.1%)
Lymphomas & hematopoietic neoplasms								
Lymphoma	7 (50%)	7 (50%)	14 (100%)	0 (0%)	0 (0%)	0 (0%)	14 (100%)	14 (2.0%)
Tumors of sellar region								
Tumors of the pituitary								
Pituitary adenoma	14 (58.3%)	10 (41.7%)	0 (0%)	24 (100%)	0 (0%)	5 (20.83%)	19 (79.17%)	24 (3.5%)
Craniopharyngioma								
Craniopharyngioma	3 (100%)	0 (0%)	0 (0%)	3 (100%)	0 (0%)	2 (66.67%)	1 (33.33%)	3 (0.4%)
Papillary craniopharyngioma	1 (100%)	0 (0%)	0 (0%)	1 (100%)	0 (0%)	1 (100%)	0 (0%)	1 (0.1%)
Unclassified tumors								
Hemangioma								
Hemangioma	1 (50%)	1 (50%)	0 (0%)	2 (100%)	0 (0%)	0 (0%)	2 (100%)	2 (0.3%)
Epithelioid hemangioendothelioma	1 (100%)	0 (0%)	0 (0%)	1 (100%)	0 (0%)	0 (0%)	1 (100%)	1 (0.1%)
Cavernous hemangioma	2 (50%)	2 (50%)	0 (0%)	4 (100%)	0 (0%)	3 (75%)	1 (25%)	4 (0.6%)
Total	322 (46.3%)	373 (53.7%)	424 (61.01%)	271 (38.99%)	59 (8.49%)	156 (22.45%)	480 (69.06%)	695 (100%)
